# Phase-contrast MRI versus numerical simulation to quantify hemodynamical changes in cerebral aneurysms after flow diverter treatment

**DOI:** 10.1371/journal.pone.0190696

**Published:** 2018-01-05

**Authors:** Sergey Sindeev, Philipp Georg Arnold, Sergey Frolov, Sascha Prothmann, Dieter Liepsch, Andrea Balasso, Philipp Berg, Stephan Kaczmarz, Jan Stefan Kirschke

**Affiliations:** 1 Department of Biomedical Engineering, Tambov State Technical University, Tambov, Russia; 2 Department of Neuroradiology, Klinikum rechts der Isar of Technical University of Munich, Munich, Germany; 3 Department of Building Services Engineering, Chemical Engineering for Paper and Packaging, Print and Media Technologies, Munich University of Applied Sciences, Munich, Germany; 4 Department of Earth and Environmental Sciences, Ludwig-Maximilian-University of Munich, Munich, Germany; 5 Department of Fluid Dynamics and Technical Flows, University of Magdeburg, Magdeburg, Germany; Technion Israel Institute of Technology, ISRAEL

## Abstract

Cerebral aneurysms are a major risk factor for intracranial bleeding with devastating consequences for the patient. One recently established treatment is the implantation of flow-diverters (FD). Methods to predict their treatment success before or directly after implantation are not well investigated yet. The aim of this work was to quantitatively study hemodynamic parameters in patient-specific models of treated cerebral aneurysms and its correlation with the clinical outcome. Hemodynamics were evaluated using both computational fluid dynamics (CFD) and phase contrast (PC) MRI. CFD simulations and in vitro MRI measurements were done under similar flow conditions and results of both methods were comparatively analyzed. For preoperative and postoperative distribution of hemodynamic parameters, CFD simulations and PC-MRI velocity measurements showed similar results. In both cases where no occlusion of the aneurysm was observed after six months, a flow reduction of about 30-50% was found, while in the clinically successful case with complete occlusion of the aneurysm after 6 months, the flow reduction was about 80%. No vortex was observed in any of the three models after treatment. The results are in agreement with recent studies suggesting that CFD simulations can predict post-treatment aneurysm flow alteration already before implantation of a FD and PC-MRI could validate the predicted hemodynamic changes right after implantation of a FD.

## Introduction

A flow-diverter (FD) is a promising tool for the treatment of wide-necked and fusiform aneurysms which has a low complication rate [1–4]. Nevertheless the prediction of the treatment success is complicated since the flow parameters which influence the clinical outcome are still not well understood. Commonly in clinical practice a post-treatment contrast agent stasis is considered to assess the flow diverting effect immediately after the FD placement. However the observed flow stasis could not be a predictor of a complete aneurysm occlusion in some cases [5]. Therefore the clinical decision-making process is largely dependent on global risk factors and on experience and qualification of the neurosurgeon or neuroradiologist [6].

A number of studies, both experimental and numerical, have been done during recent years to find reliable hemodynamic parameters that could evaluate the FD performance and predict the clinical outcome [7–9]. Tsang et al [10] found a correlation between a significant change in turnover time after stenting and aneurysm occlusion, suggesting that it could be one of the criteria to evaluate the flow diversion effect and clinical success. Similarly Janiga et al [11] used the turnover time (residence time) to analyze different treatment scenarios and select an appropriate stent model and position leading to maximal increase in residence time.

Additionally some other parameters taking into account the flow pattern inside an aneurysm sac could be considered. In an experimental study by Balasso et al [12] the FD performance was assessed by complex analysis of a change of maximum velocity at the inflow zone, in the dome and at the outflow zone of the aneurysm. Moreover in the study by Suzuki et al [13] the relative reduction of the spatial-averaged and maximum velocity within the aneurysm sac as well as the maximum wall shear stress (WSS) were considered to analyze the flow reduction effects after the treatment. Another alternative, proposed by Lieber et al, is an assessment of the stenting effect according to kinetic energy reduction [14] and change of the vorticity inside an aneurysm [15].

Despite recent achievements of numerical and experimental methods for evaluation of the FD performance, their application in clinical practice is still constrained due to their complexity and time restrictions. For example, evaluation of post-treatment blood flow immediately after the stent placement using CFD methods is problematic, since typical numerical simulation with FD could take several weeks depending on the size of the computational mesh. In the same time several feasibility studies showed that MRI could serve as a tool for the clinical assessment of cerebral hemodynamics which could rapidly evaluate the intra-aneurysmal flow field with sufficient precision [16–18]. However Pereira et al [19] reported technical issues in the measurement of low velocities and the need for more validation work in a feasibility study regarding post-treatment evaluation of blood flow in aneurysms after FD placement using MRI. Further MacDonald et al [20] assessed the pre- and postoperative hemodynamic parameters for a giant cerebral aneurysm and found that the intra-aneurysmal pressure derived from MRI measured velocity field differed only by 6.1% from the pressure measured by invasive techniques. However there is a lack of studies which evaluate hemodynamic changes in cerebral aneurysms after a FD placement using both experimental and numerical methods.

The purpose of this work was to quantitatively study the distribution of hemodynamic parameters in patient-specific models of cerebral aneurysms before and after treatment with flow diverters using PC-MRI and CFD and to correlate these results with the clinical outcome.

## Materials and methods

### Ethics committee approval

As our experiments were conducted retrospectively with permanently anonymized patient data, our local ethics committee deemed the study exempt from the requirement for approval.

### Clinical cases

Twenty three patients treated with a FD at the department of Neuroradiology at the Klinikum rechts der Isar between 2008 and 2014 were screened for availability of pre-treatment high spatial resolution 3D angiographic data. These datasets were available for three patients, who were included in this study and subgrouped regarding their clinical treatment outcome. This study was HIPAA compliant and in line with the local ethical and legislative requirements. Patient consent was not required due to the fully anonymous retrospective analysis.

Group A consisted of one patient (A01) with a clinically successful treatment. This aneurysm showed no perfusion 6 months after FD placement. Group B included two patients (B01, B02) who showed persistent perfusion of the aneurysm for more than 6 months after treatment. A detailed information about the studied cases is presented in Table 1.

**Table 1:** Clinical outcome for the treated aneurysms.

Case	Sex	Age	FD used	Clinical outcome
B01	M	30	**In vivo:** SILK 4.5x60 mm Leo 4.5x40 mm **In vitro:** DERIVO 5.0x50 mm DERIVO 4.5x40 mm	Angiography after 6 months showed reduced but still existing perfusion of the aneurysm. Occlusion was achieved one year later after additionally using 24 platin coils.
B02	M	58	**In vivo:** Pipeline 4.5x20 mm **In vitro:** DERIVO 5.5x20 mm	Angiography after 6 month showed no delay in inflow and only a subtle delay in outflow. The aneurysm was later treated with an additional FD which led to occlusion of the aneurysm.
A01	F	48	**In vivo:** SILK 3.5x25 mm **In vitro:** DERIVO 3.5x25 mm	Angiography after 7 month showed no perfusion of the aneurysm dome.

### Experimental phantoms

Realistic silicone phantoms were produced for each patient. The patient’s pre-intervention CT angiographic data was used to segment the aneurysm and adjacent vessels to generate STL models. These STL models served as template for a high precision 3D wax printer. The silicone Elastosil 601 was molded around the wax which was later removed using a dissolvent. For each case 2 silicone phantoms were manufactured by Acandis (Pforzheim, Germany).

**Fig 1:**
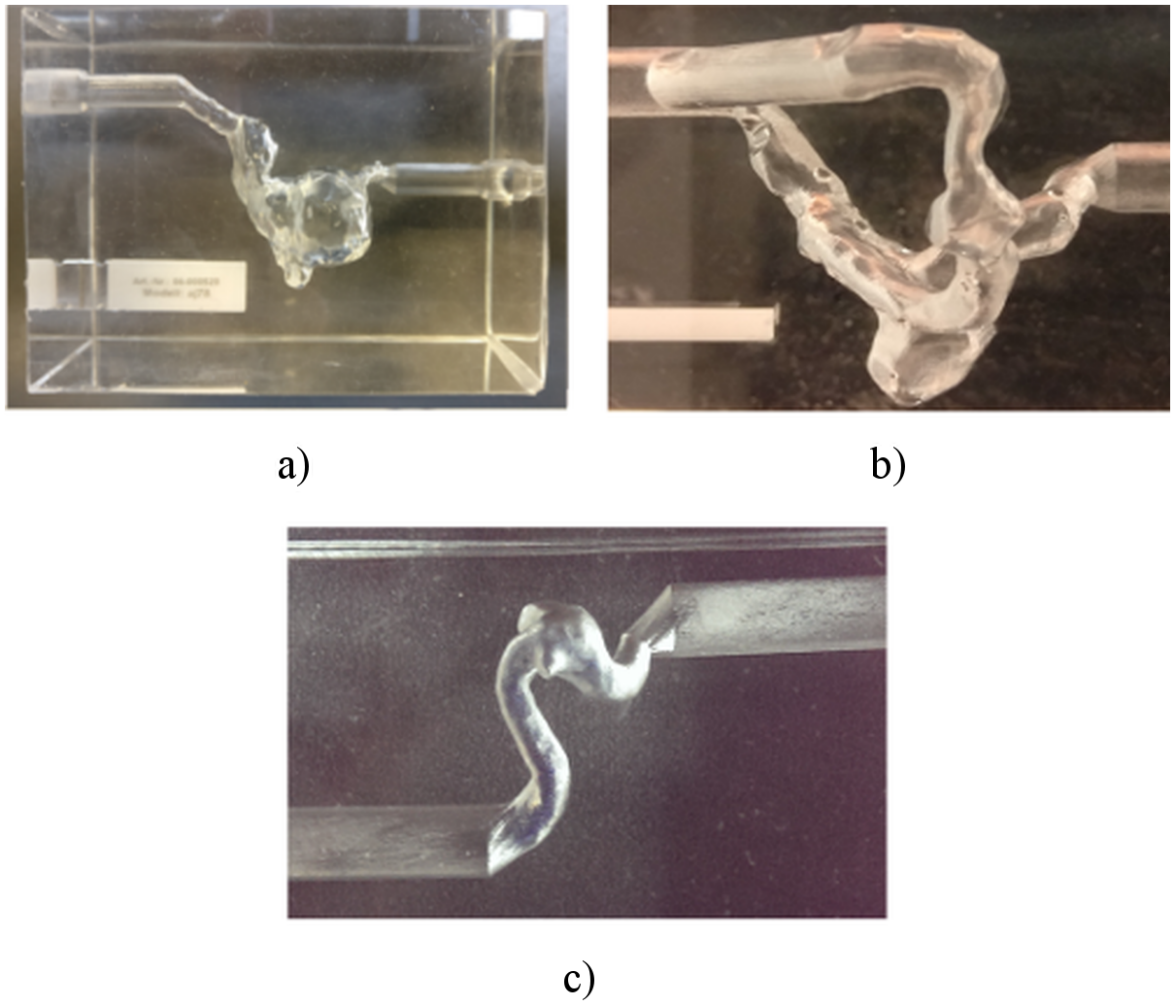
Aneurysm phantoms used for hemodynamics studies. a)—B01; b)—B02; c)—A01.

One phantom of each aneurysm was used to study preoperative hemodynamics. The other one was used to study hemodynamic changes after the placement of the corresponding FD. The aneurysm phantoms are presented in Fig 1. These phantoms were used for MRI flow measurements. The dimensions of the studied phantoms are presented in Table 2.

**Table 2:** Dimensions of the aneurysms. All dimensions are in *mm*.

№	Case	Group	Neck	Length	Width	Height	Aspect ratio
1	B01	B	19	19	16	20	1.1
2	B02	B	8	9	9	10	1.2
3	A01	A	5	6	6	6	1.1

### Experimental setup

**Fig 2:**
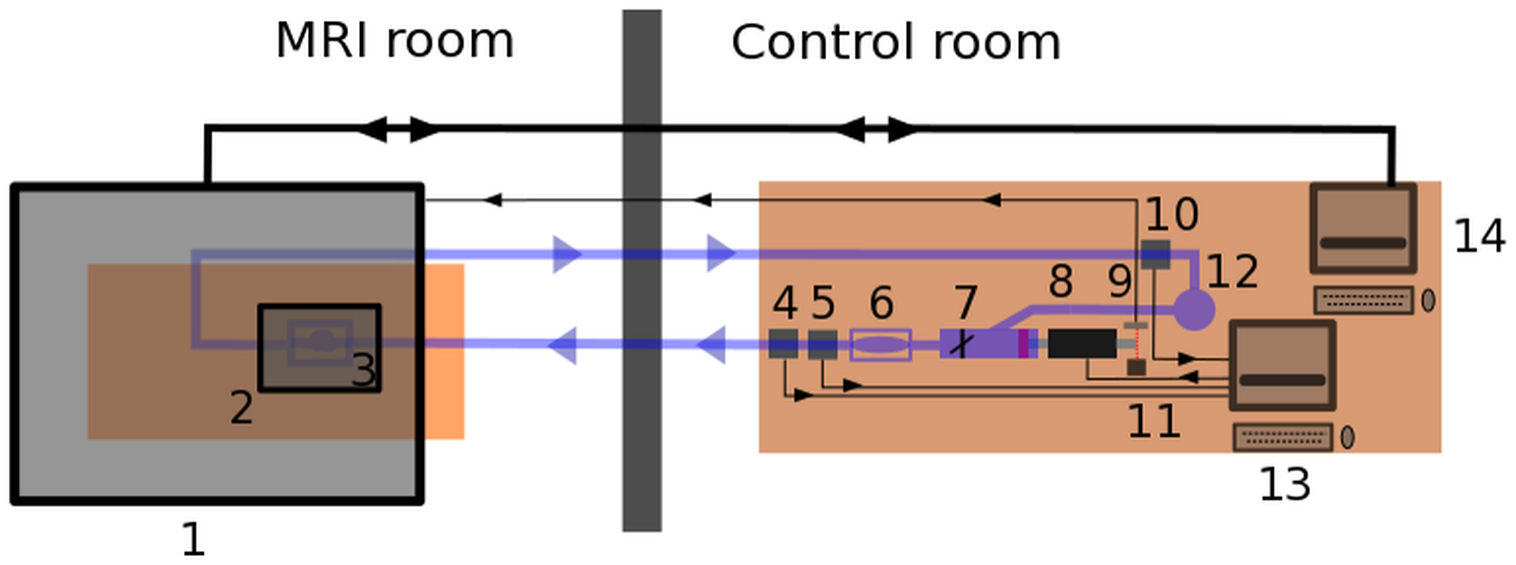
Scheme of experimental setup. 1—MRI Philips Ingenia 3T; 2—MRI handwrist-coil; 3—aneurysm phantom; 4—inlet pressure sensor; 5—flow sensor; 6—compliance chamber; 7—valve; 8—piston pump; 9—photo diode; 10—outlet pressure sensor; 11—LED; 12—pressure chamber; 13—measurement computer; 14—MRI computer.

The measurement setup (Fig 2) included a computer controlled piston pump (8). The valve (7) mimicked the aortic valve. The compliance chamber (6) simulated the windkessel function of the aorta. The experimental fluid was a 58% aqueous glycerol mixture, which exhibits Newtonian fluid behavior. The flow rate (5), the inlet pressure (4) and the outlet pressure (10) were measured with the corresponding sensors and recorded by the measurement computer (13). The aneurysm phantom (3) was placed inside a MRI hand wrist coil (2) which allowed for better signal to contrast ratio and thus spatial resolution. The MRI (1) was synchronized with the piston pump (8) using a photo diode (9) which provided a trigger signal. The pressure chamber (12) was used to provide a constant diastolic pressure. The realistic inlet flow rate of 7 *l*/*h* was used, which corresponded to the average velocity of 0.25 *m*/*s* for B01, 0.14 *m*/*s* for B02 and 0.19 *m*/*s* for A01 at the expanded inlet segment during the systolic peak.

The measurement protocol is available as supporting information file (S1 Protocol) or at protocols.io (http://dx.doi.org/10.17504/protocols.io.kekctcw).

### MRI settings

A 3T Philips Ingenia (Philips Healthcare, Netherlands) was used as MRI velocity measuring system. The aneurysm phantoms were placed inside a hand wrist coil to maximize spatial resolution and signal-to-noise ratio (SNR). A 4D phase contrast sequence with a multi-shot 3D Gradient-Echo sequence with a turbo-factor of 5 without SENSE was used for image acquisition. The acquisition parameters were set to: TE = 2.9 *ms*, TR = 6.1 *ms*, *α* = 10°. Flow in all 3 spatial directions (ap, rl, cc) were acquired consecutively in one single scan. Total scan duration was 30:44 min.

The quantitative velocity field was reconstructed with corrections for magnetic field inhomogeneities by static measurements. The spatial resolution was RLxAPxFH = 0.53x0.53x0.60 *mm*^3^ with a matrix size of 112x112x94 voxels. The temporal resolution was 71 *ms*. After inspection of the CFD velocity data velocity encoding level (VENC) was set to 100 *cm*/*s* for each spatial direction to prevent aliasing in the aneurysm region. Partial volume effects close to the vessel wall were neglected as the drop in magnitude signal for all cases with and without FD was steep and abrupt.

### CFD settings

The Navier-Stokes equations for incompressible fluid were utilized for CFD simulations. The inlet velocity curves were obtained from the data of the MRI velocity measurements. The fluid density was set to *ρ* = 1141 *kg*/*m*^3^ which corresponds to the density of the experimental fluid. A Newtonian model was used to represent the fluid behavior. The viscosity was set to *η* = 4.1 *mPa* ⋅ *s*, which corresponded to the used experimental fluid.

For the numerical studies the geometrical models of the aneurysms were created using corresponding STL-models of the silicone phantoms. The geometrical models of Acandis DERIVO FDs were created according to the manufacturer specifications. Fast virtual stenting technique was used for the virtual deployment of the FDs in the aneurysm models. Here, nominal diameters, initial lengths, strut diameters as well as strut angles were respected. Hence, arbitrary configurations can be reproduced. This explicit method considers the individual stent pores and is therefore clearly superior compared to simplified approaches such as the assumption of a porous medium to account for flow-diverting effects. The virtually deployed FDs were visually compared with the available in-vitro deployments and showed sufficient correlation. A detailed description of the virtual stenting technique was presented in the studies by Berg et al [21, 22]. Furthermore, a validation of the fast virtual stenting approach can be found in the study by Janiga et al. [11].

The hexahedral computational meshes were generated using snappyHexMesh tool from OpenFOAM CFD Toolbox (CFD Direct, Caversham, England). Additional refinement of the cells around the FD was used to properly represent the minimal elements of FD braid. A mesh independence test showed that generated meshes were sufficient to capture all significant features of the flow pattern in the studied aneurysms. Five cardiac cycles were simulated to omit the initial perturbations of the flow field. The results for the last cardiac cycle were used for analysis.

### Analysis of hemodynamic parameters

The moment of systolic peak was selected to analyze the hemodynamic parameters in the aneurysms. This moment was used since hemodynamic parameters reach their maximum values at this time. The flow pattern in the central cross-section of each aneurysm was analyzed in detail as well as the WSS distribution, streamlines, kinetic energy and recirculation. Additionally, the hemodynamic changes after FD placement were analyzed and correlated with the available clinical outcome.

The WSS was computed to analyze the shear stress distribution over the aneurysm sac. To derive the WSS from the MRI-measured velocity field, the wall shear rate was computed, which then was multiplied by dynamic viscosity *η*. The normalized WSS (nWSS) was used to compare the WSS distribution between CFD and MRI:

$$\begin{array}{c} nWSS=\frac{WSS(x,y,z)}{WSS_{max}}, \end{array}$$

where *WSS*(*x*, *y*, *z*) is the WSS magnitude at the point with coordinates (*x*, *y*, *z*); *WSS*_*max*_ is the maximum WSS magnitude over the aneurysm sac.

To evaluate a kinetic energy in the aneurysm sac we used a method proposed by Seong et al. [14, 23], according to which a measure of the intra-aneurysmal kinetic energy *E* could be estimated as a sum of squares of velocity magnitudes *u*_*i*_ at every *i*-th measured point (*x*, *y*, *z*) in the aneurysm sac, i.e.:

$$\begin{array}{c} E=\sum_{i=1}^{N}u_{i}^{2}\left(x,y,z\right), \end{array}$$ (1)

where *N* is a number of measured points.

Mean kinetic energy over the cardiac cycle was employed to quantify hemodynamic changes after the FD placement, which is an average of sum of instantaneous kinetic energies over the cardiac cycle. Since CFD results have a much higher spatial and temporal resolution a comparison of absolute values of kinetic energy (1) between MRI and CFD is not valid. Therefore only relative kinetic energy reductions *E*_*R*_ for CFD and MRI results were considered in this study:

$$\begin{array}{c} E_{R}=\frac{E_{noFD}-E_{FD}}{E_{noFD}}\cdot 100\%, \end{array}$$

where *E*_*noFD*_ and *E*_*FD*_ are the intra-aneurysmal kinetic energies before and after FD placement respectively.

Another hemodynamic parameters used to assess FD performance were peak and mean recirculation [15]. The instantaneous recirculation *R* could be obtained by computing vorticity *ω* of the intra-aneurysmal velocity field:

$$\begin{array}{c} \omega =\nabla \times u, \end{array}$$

$$\begin{array}{c} R=\;\int \int \;\omega \cdot n\;dA \end{array}$$ (2)

The mean recirculation *R*_*m*_ could be found by integrating Eq (2) over the cardiac cycle:

$$\begin{array}{c} R_{m}=\frac{1}{T}\cdot \int_{0}^{T}|R|dt, \end{array}$$

where *T* is the length of the cardiac cycle.

## Results

### Preoperative analysis

**Fig 3:**
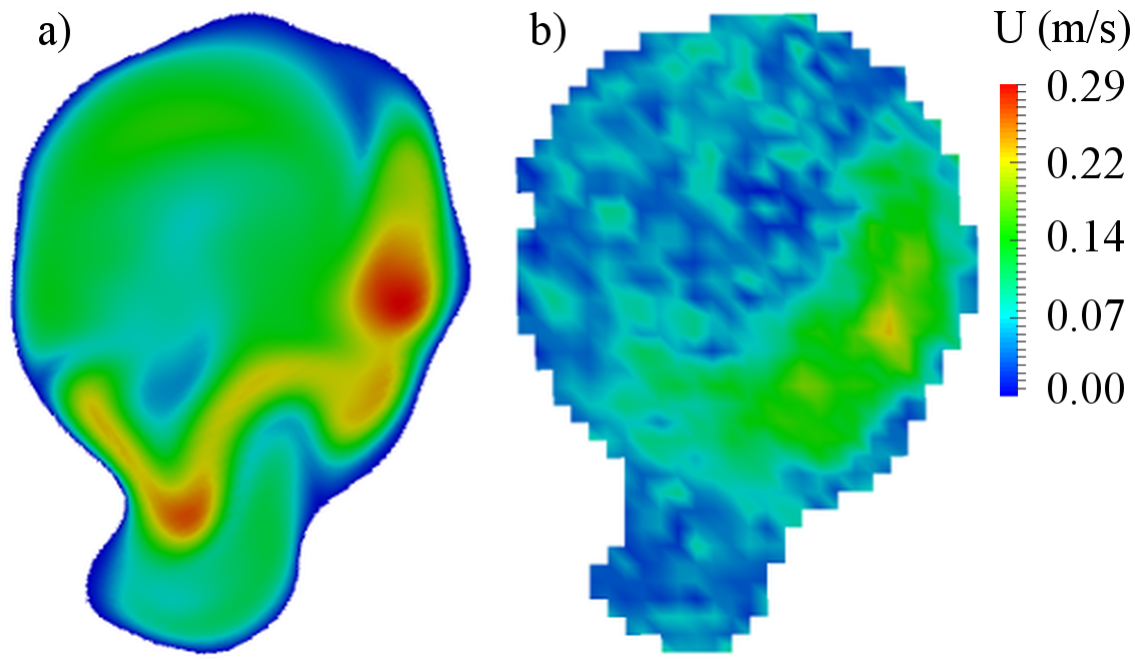
Preoperative velocity distribution for central cross-section of B01 aneurysm. a) CFD simulation; b) MRI measurement.

**Fig 4:**
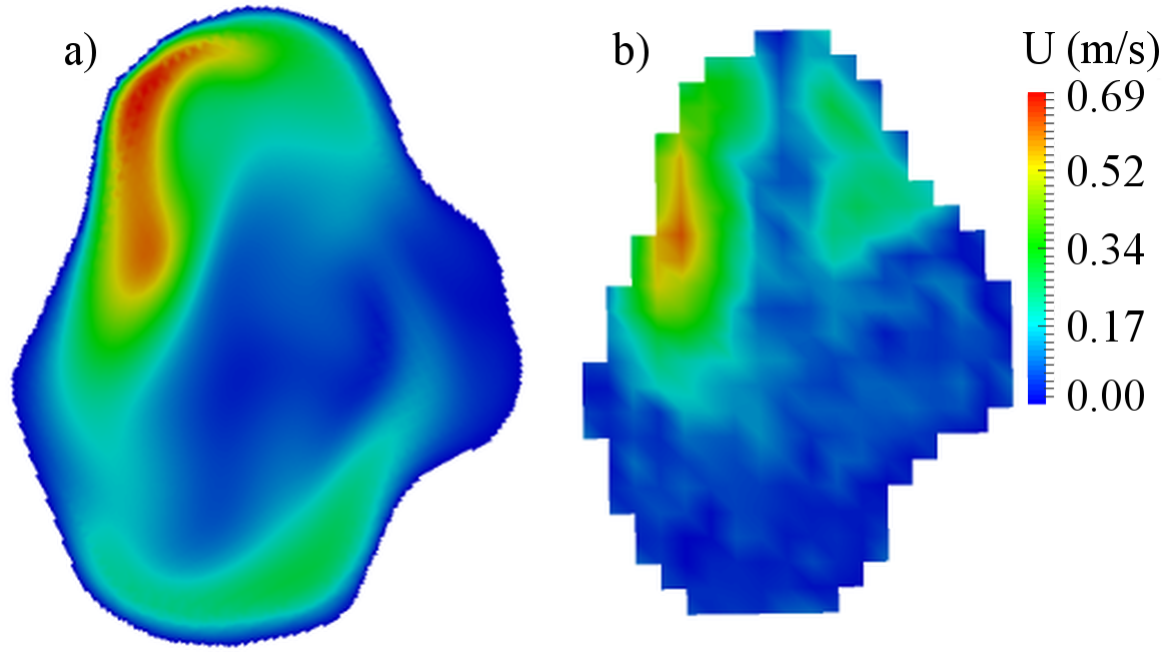
Preoperative velocity distribution for central cross-section of B02 aneurysm. a) CFD simulation; b) MRI measurement.

**Fig 5:**
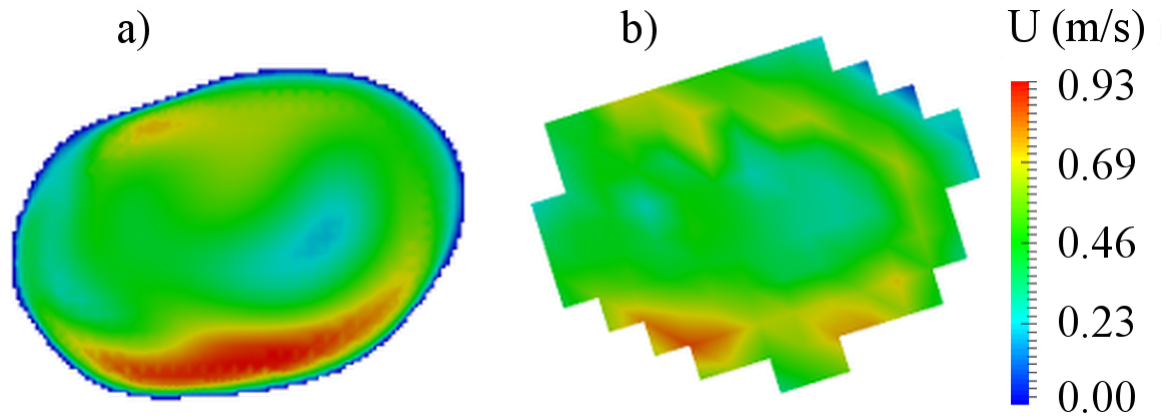
Preoperative velocity distribution for central cross-section of A01 aneurysm. a) CFD simulation; b) MRI measurement.

The central cross-sections of the aneurysm models were used to analyze the flow pattern in the aneurysm sac. The cross-sections are presented in Figs 3, 4 and 5 for cases B01, B02 and A01 respectively. The CFD simulations and MRI measurements predicted a similar main flow pattern for the all three cases. The region which belongs to the aneurysm sac itself, excluding the parent artery, was used to compute an average intra-aneurysmal blood flow. It was done in order to correctly compare the average intra-aneurysmal velocity before and after the treatment. The obtained average intra-aneurysmal velocity is presented in Table 3. The average difference between CFD and MRI results was 9.47% for B01; 6.1% for B02 and 6.21% for A01.

**Table 3:** Hemodynamic parameters before and after the FD placement. *noFD*—before the treatment; *FD*—after the treatment; *U*—an average intra-aneurysmal velocity during the systolic peak (*m*/*s*); *E*—mean kinematic energy (*m*^2^/*s*^2^); *R*—mean recirculation (*mm*^2^/*s*).

	noFD						FD					
	CFD			MRI			CFD			MRI		
	U	E	R	U	E	R	U	E	R	U	E	R
B01	0.089	806	92	0.081	2.8	87	0.039	395	16.56	0.040	1.32	18.27
B02	0.075	929	89	0.071	14.9	79	0.045	724	22.25	0.063	11.8	30.02
A01	0.416	2818	74	0.443	18.4	81	0.087	592	11.1	0.076	3.5	10.53

**Fig 6:**
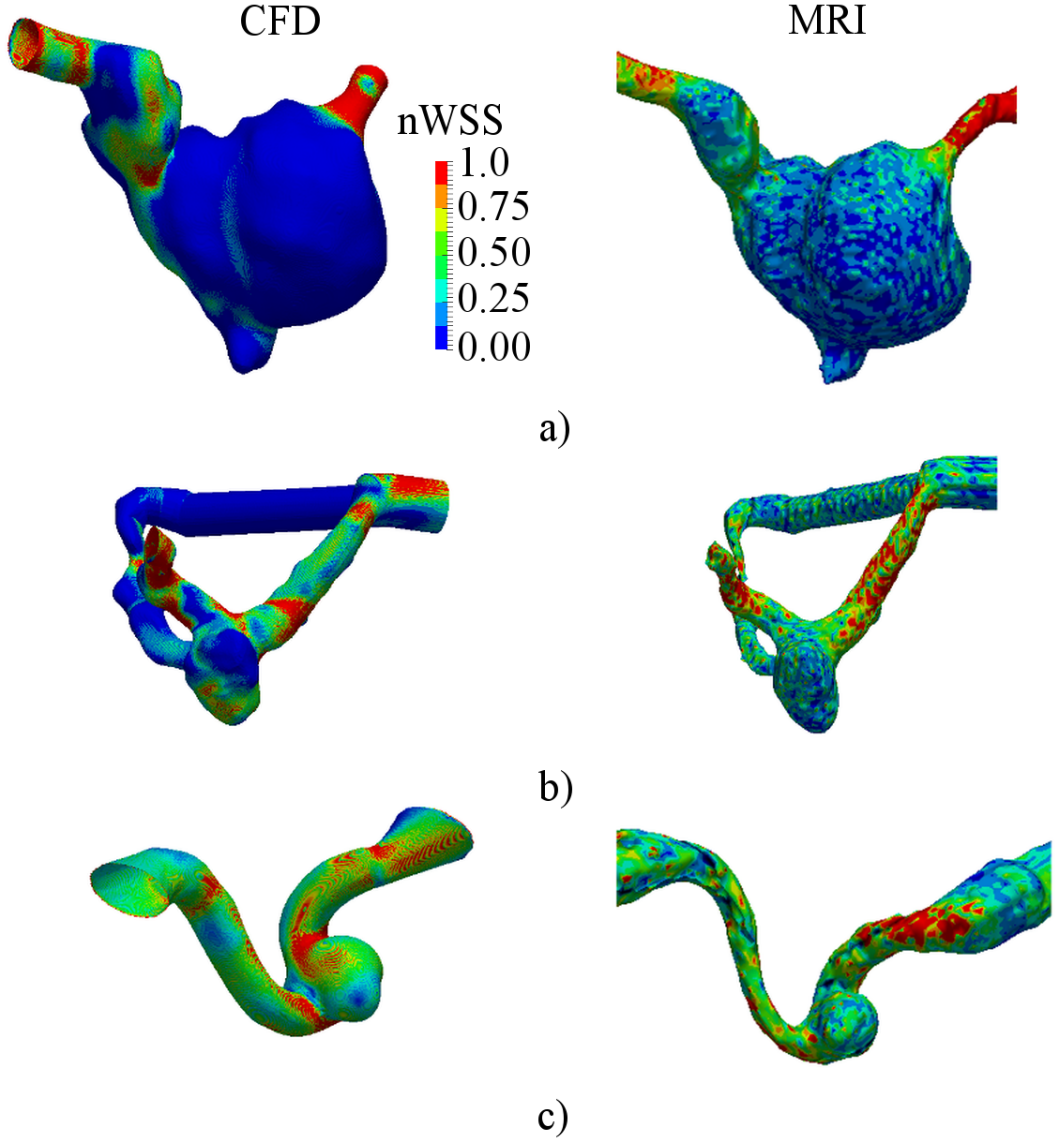
Pre-operative distribution of normalized wall shear stress in aneurysm models. a)—B01; b)—B02; c)—A01.

The nWSS is shown in Fig 6 both for CFD and MRI. Similar general nWSS distributions were observed for the aneurysm models, but detailed variations in WSS were not adequately revealed by MRI. Regions of high nWSS (≥ 0.75) were found at the proximal and distal segments of the parent artery. Contrary, the aneurysm sac and aneurysm bleb were characterized by low values of nWSS (≤ 0.25). However the distribution of nWSS for MRI was influenced by noise and low spatial resolution.

**Fig 7:**
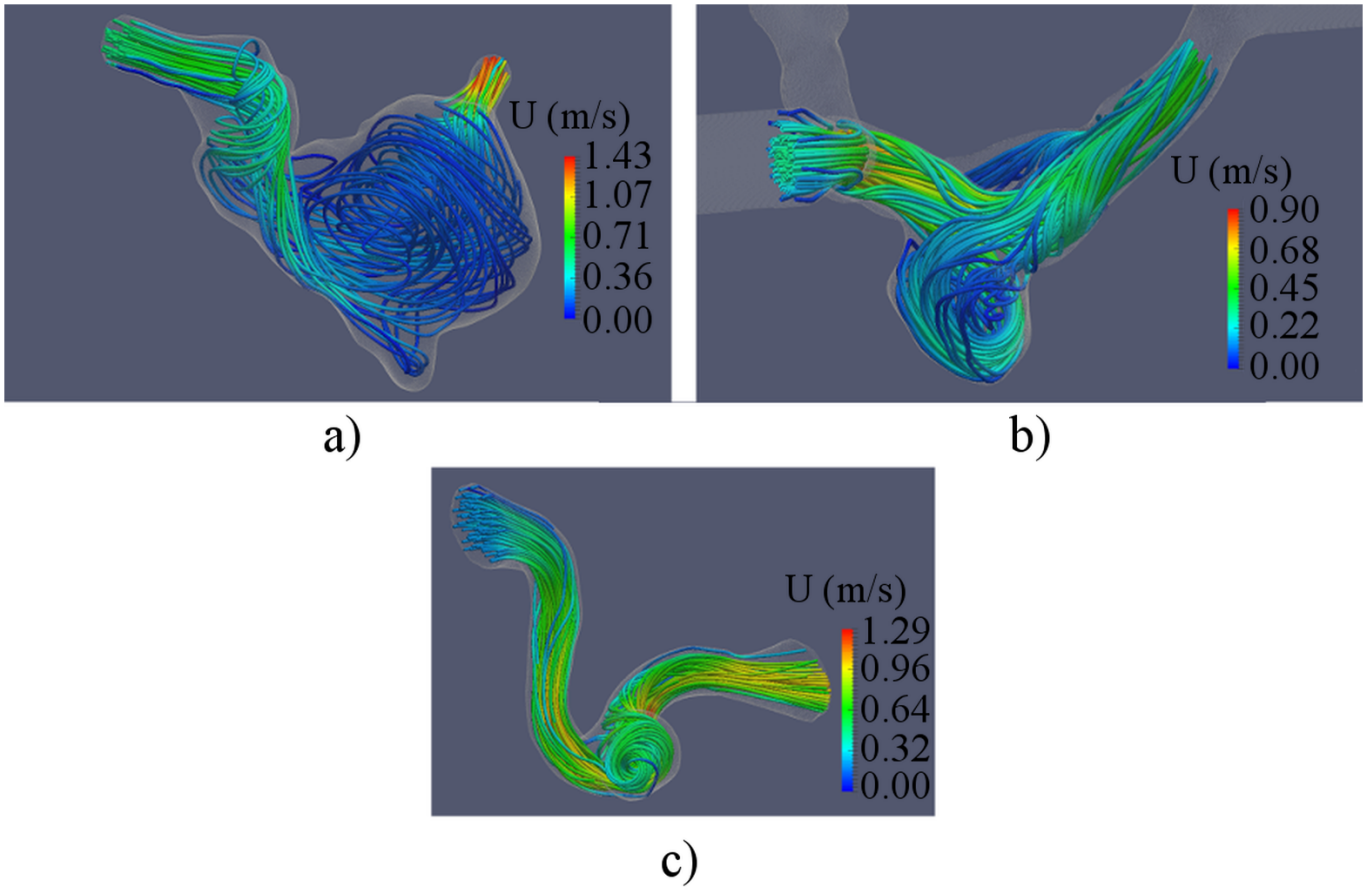
Streamlines in cerebral aneurysms before flow-diverter placement. a)—B01; b)—B02; c)—A01.

Streamlines were computed from CFD data to detect vortices in the aneurysm sac. The computed streamlines for the studied aneurysms are presented in Fig 7. The preoperative blood flow in the aneurysms was characterized by the presence of a vortex in all three models. The swirling flow in the aneurysm sac produced the region of low velocities in the center of the aneurysms.

### Postoperative analysis

**Fig 8:**
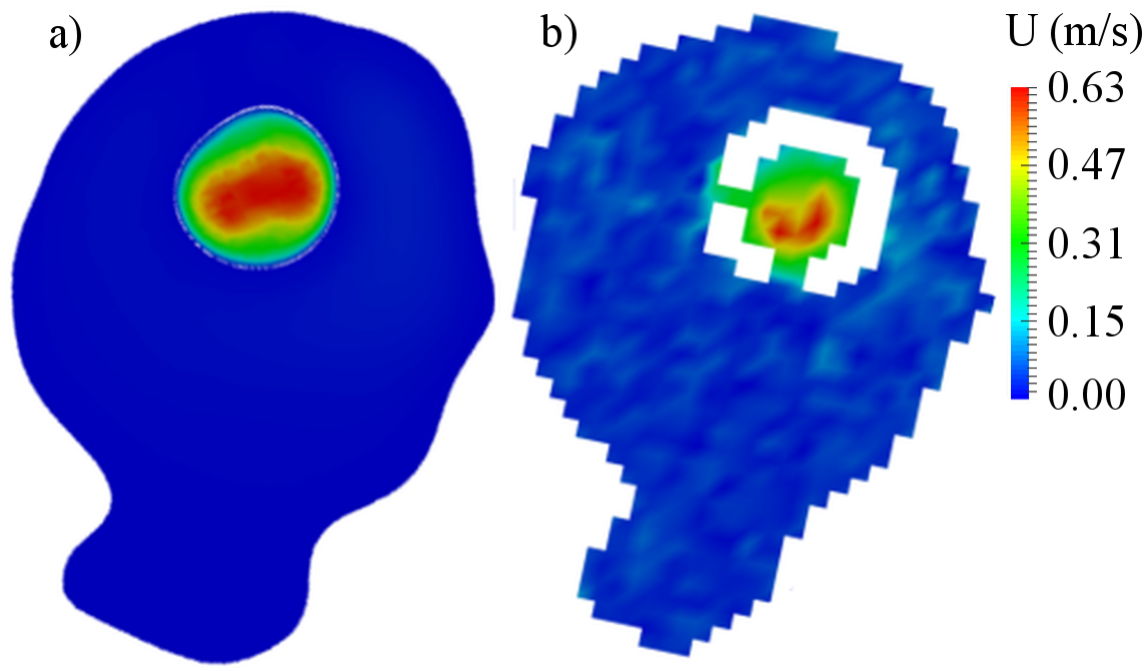
Velocity distribution for central cross-section of B01 aneurysm after flow-diverter placement. a) CFD simulation; b) MRI measurement.

**Fig 9:**
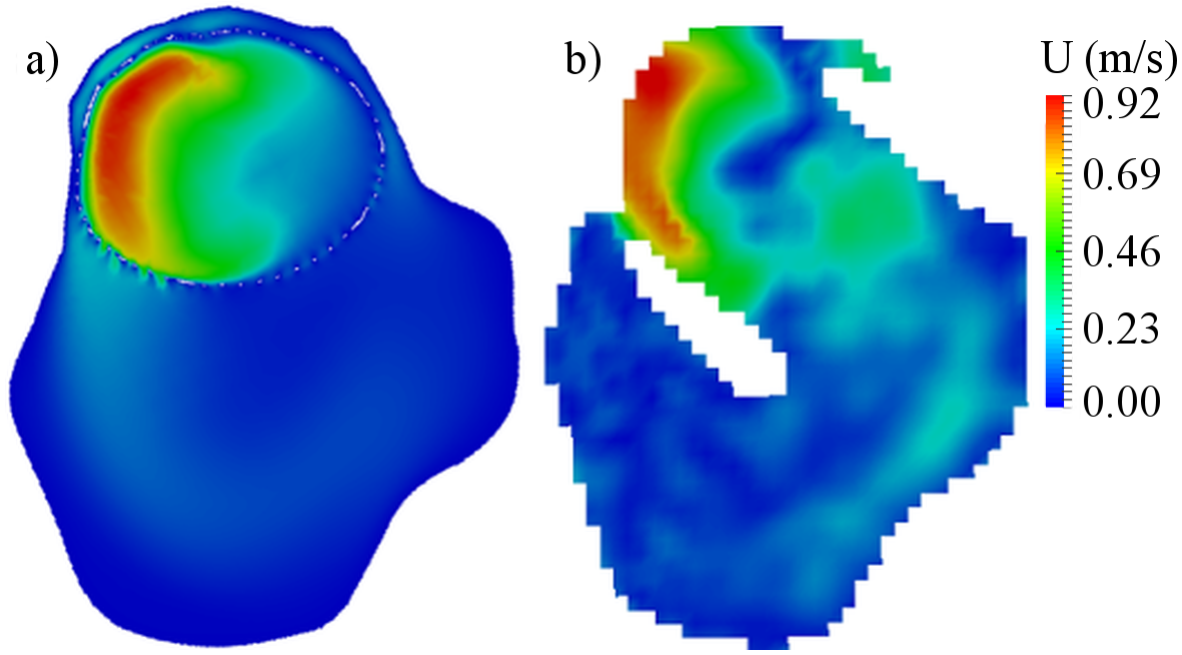
Velocity distribution for central cross-section of B02 aneurysm after flow-diverter placement. a) CFD simulation; b) MRI measurement.

**Fig 10:**
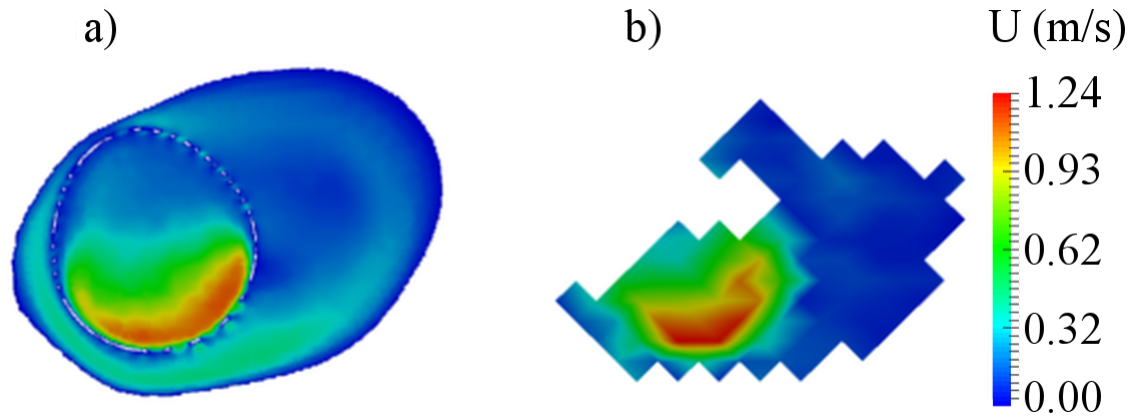
Velocity distribution for central cross-section of A01 aneurysm after flow-diverter placement. a) CFD simulation; b) MRI measurement.

The flow-patterns in the aneurysms were decidedly changed after FD placement. The central cross-sections for the treated aneurysms are presented in Figs 8, 9 and 10 for cases B01, B02 and A01 respectively. Both CFD and MRI showed a general similarity of flow-patterns for the treated cases. However the presence of metallic braiding of the FD led to non-visualizable regions in the central cross-sections for MRI data. The maximum velocity region was jailed by the braid of FD, which led to reduction of blood flow in the aneurysm sac. The obtained reduced intra-aneurysmal velocities for studied models are presented in Table 4. The observed flow reduction was about 52%, 28% and 80% for cases B01, B02 and A01 respectively.

**Table 4:** Relative change of hemodynamic parameters after the FD placement.

	Δ*U*, %		Δ*E*, %		Δ*R*, %	
	CFD	MRI	CFD	MRI	CFD	MRI
B01	56	51	51	53	82	79
B02	39	11	22	25	75	62
A01	79	83	79	81	85	87

**Fig 11:**
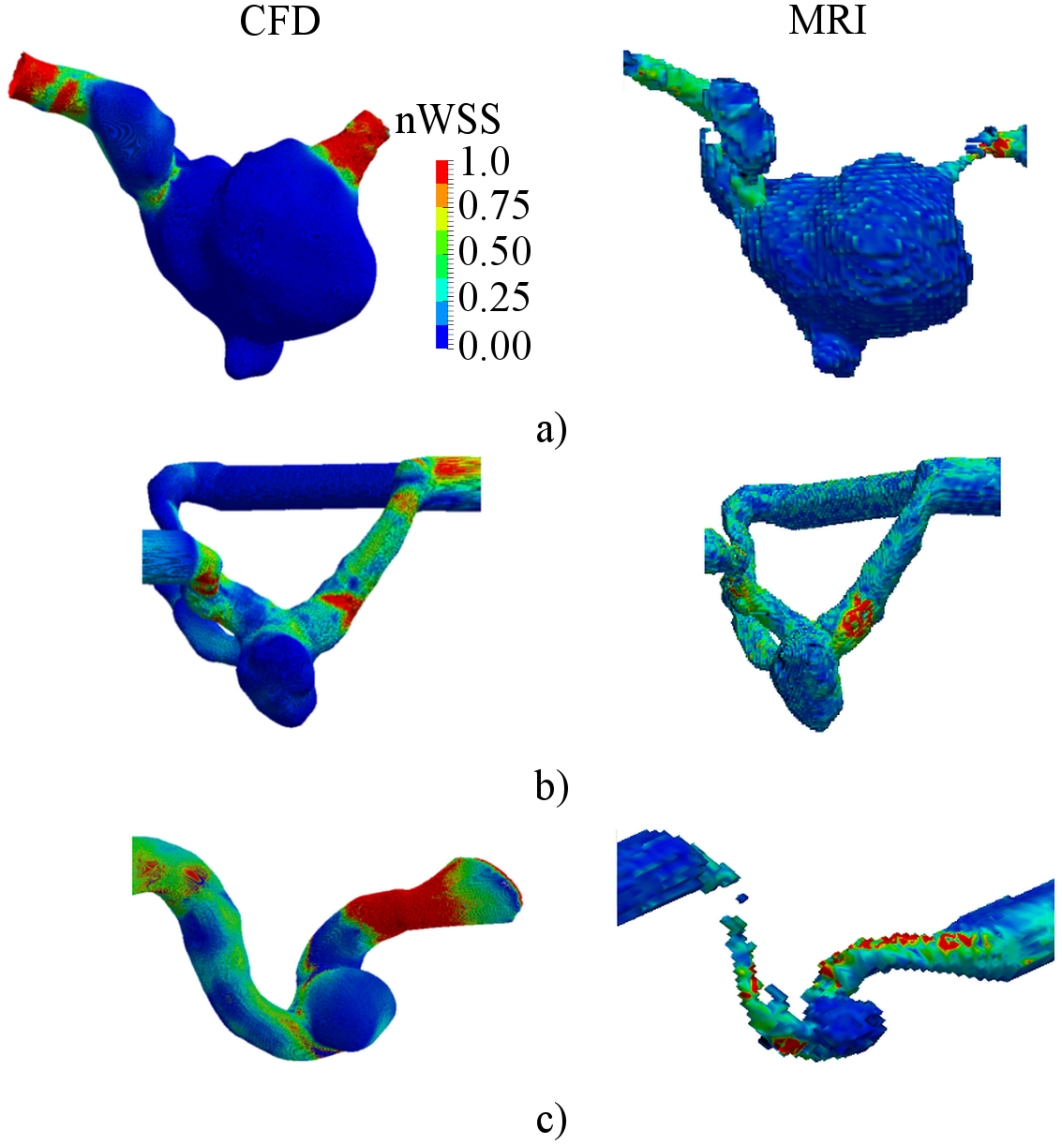
Distribution of normalized wall shear stress after flow-diverter placement. a)—B01; b)—B02; c)—A01.

**Fig 12:**
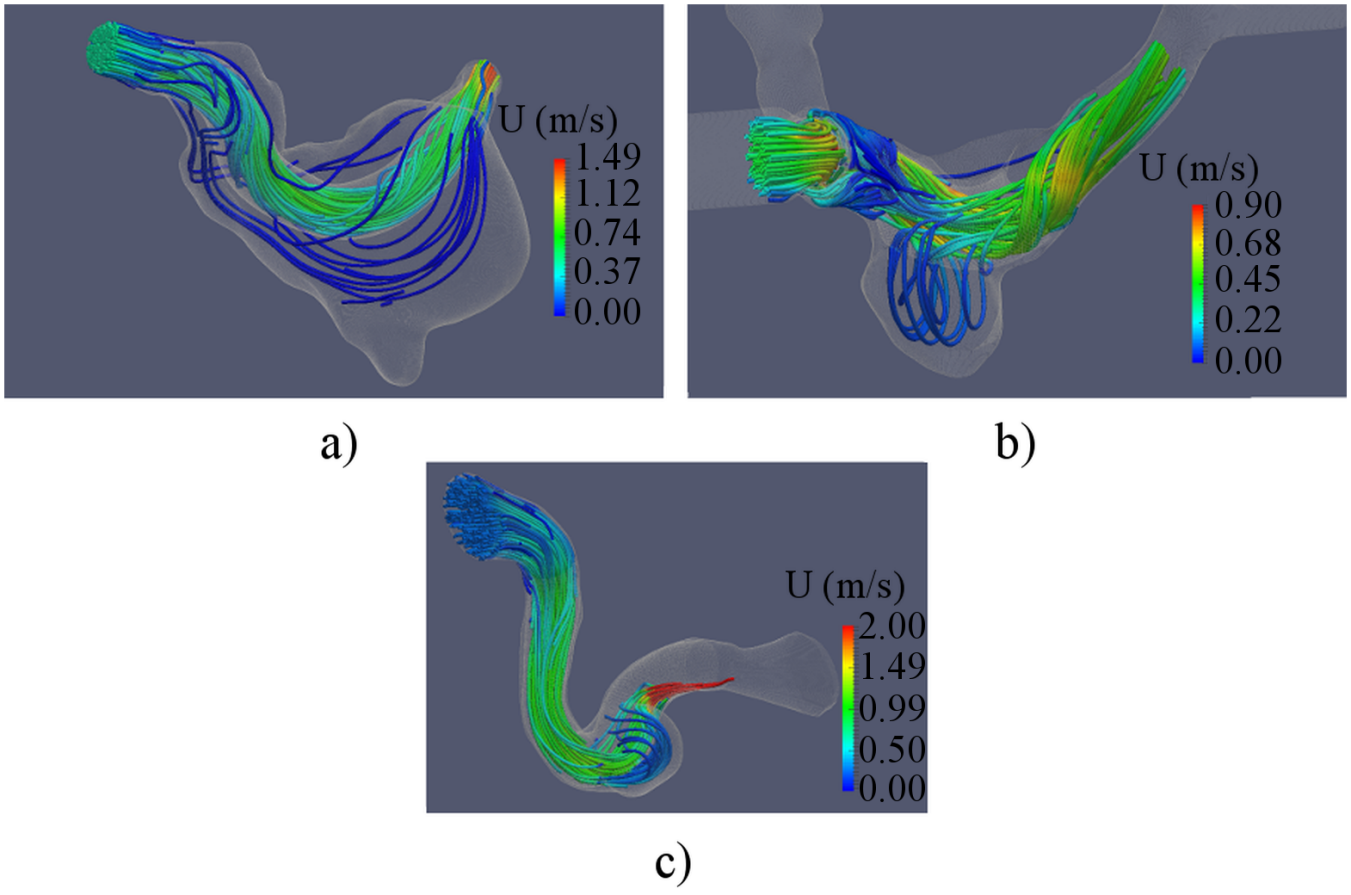
Streamlines in cerebral aneurysms after flow-diverter placement. a)—B01; b)—B02; c)—A01.

Also nWSS was computed to determine the changes in WSS distribution after treatment. No significant changes in distribution pattern were found (Fig 11). However the average WSS magnitude was decidedly reduced in all three cases. Streamlines were computed to determine the changes of flow patterns after FD placement. The computed streamlines are presented in Fig 12. The major part of the flow was redirected along the FD, while the intra-aneurysmal flow was decidedly reduced. The vortex in the aneurysm sac disappeared in all three models.

## Discussion

In the present study we analyzed the hemodynamic changes in cerebral aneurysms of 3 patients before and after FD treatment and correlated them with their known clinical outcome. Similar velocities and flow patterns were obtained for both CFD and MRI methods while substantial differences were found for calculated WSS. We observed a larger flow reduction for the successfully treated aneurysm, while a smaller reduction was found for the two cases where additional treatment was needed for occlusion.

A relative flow reduction is considered a major parameter correlated with the clinical outcome of the treatment. Xiang et al [24] found a reduction of average aneurysmal velocity by 76% for the aneurysm which was occluded within the first three months and a reduction by 40% for the aneurysms that occluded after six months. It was proposed that the post-treatment reduction of average intra-aneurysmal velocity could be correlated with aneurysm occlusion. Also the flow reduction is used to evaluate the flow-diversion efficiency and is considered as the objective function for optimization of FDs [25]. However Cebral et al [26] demonstrated that the reduction of intra-aneurysmal velocity was not sufficient for a successful treatment of aneurysms with a FD. Despite the decreased velocity in the aneurysm sac, an increase of intra-aneurysmal pressure after the FD placement was observed for the ruptured cases.

The results of the present study are in agreement with recent studies, which proposed that for a successful treatment with a FD the flow reduction should be at least one-third of the preoperative state [27–29]. On the other side, a flow reduction of 52% was observed for the the giant fusiform aneurysm, where an immediate clinical success was not achieved. It demonstrates that the clinical outcome is determined not only by the relative flow reduction, but it depends on a complex set of parameters, which characterize the geometry of the aneurysm [30, 31], the hemodynamics in the aneurysm sac [32], the aneurysm wall structure and blood coagulation properties [33–35].

The preoperative state of all three aneurysms was characterized by the presence of a vortex in the aneurysm sac. A zone of low velocities was observed in the center of the vortex, which could contribute to thrombus formation [36–38]. The placement of FD led to significant changes in flow-patterns for the all three cases. No vortex was observed in the aneurysms sac after the treatment. The observed relative reduction of recirculation in the studied cases is in agreement with results of Lieber et al [14] suggesting that clinical success cannot be determined by using only a recirculation as a criterion.

The virtual deployment of a FD enables a precise description of stent-induced hemodynamics at no risk for the individual patient. In the frame of this study, a fast virtual stenting approach was chosen based on Janiga et al. [39]. Its advantage clearly lies in the clinical applicability, since realistic deformations are performed within seconds. Furthermore, the complete geometric appearance of the stent (e.g., each single stent strut and individual pores) is considered. In contrast, other approaches reported in literature are either highly computationally expensive or over-simplify those devices due to the consideration of porous media. In this regard, the recent virtual FD deployments are well suited for the comparisons with the real stentings of this study.

WSS distribution is another important parameter which is commonly analyzed for evaluation of pre- and postoperative hemodynamics in cerebral aneurysms. There is still no clear theory whether high or low WSS values are prone to aneurysm growth and rupture [40–42]. However in some cases a correlation of high WSS with the rupture of saccular aneurysms is observed [9, 43]. In our study only the distribution of nWSS was considered, since a precise calculation of WSS using the PC-MRI measurements was problematic due to the lack of spatial resolution, especially close to the aneurysm wall, which correlates with results reported by Petersson et al [44]. Therefore the absolute values of WSS were different for CFD and MRI results and small alterations in WSS could not be revealed by MRI, however the distribution of normalized WSS showed a similar pattern for both methods, which is in agreement with the results of the recent study by Cibis et al [45].

This study has some limitations. A Newtonian fluid was used for investigation of the intra-aneurysmal hemodynamics. However recent studies showed that non-Newtonian fluid behavior could play a significant role in distribution of hemodynamic parameters in the aneurysm, especially at zones of recirculating flow [46]. The use of rigid phantoms also could lead to overestimation of hemodynamic parameters such as peak velocity magnitude and WSS [47]. Additionally no patient-specific boundary conditions were available for the studied cases, which could have a substantial effect on velocity calculations [48, 49]. However, the comparison of CFD and MRI should not be affected by these shortcomings as similar parameters were used for both methods. For better comparison within our simulations, we implanted similar FDs (DERIVO) in all the different models. As sizing of the in-vitro FD was determined based on an angiography of the respective phantom, slight differences in FD dimensions occurred compared to the patients. This may limit the comparability with the in-vivo cases, however little differences may be expected from different manufacturers and clinical corresponded well to our experimental studies. Furthermore, only a single successful patient and two unsuccessful ones were considered, limiting the generalizability of obtained individual results to other aneurysms with anatomic/geometric variability due to the limited sample size of selected patients. Future studies will address the listed limitations.

## Conclusion

In the present work we quantitatively studied the hemodynamics in three patient-specific aneurysms before and after FD treatment using both experimental and numerical methods. For preoperative and postoperative distribution of hemodynamic parameters similarity between results obtained by CFD simulations and MRI velocity measurements was shown. In cases without aneurysm occlusion after FD placement a flow reduction of about 30-50% was found, while for the clinically successful case the flow reduction was 80%. These results are in agreement with recent studies of post-treatment complications after FD placement. Both CFD and MRI methods could be used for evaluation of cerebral hemodynamics before and after treatment and could be used complementary in supporting a physician during an intervention planning. While CFD may be advantageous in a pre-treatment planning to predict the hemodynamic changes after a FD implementation, in-vivo MRI measurements are not dependent on virtual stenting and thus could assess the blood flow right after FD deployment.

While velocity reduction is clearly one aspect of the mechanism of action, it is not solely responsible for treatment success. The treatment result is more complicated and likely involves an interaction between velocity reduction, hemodynamic changes and coagulation. While no simulation can realistically incorporate all of these variables due to many unknowns, MRI measurements could provide velocity information that may factor into treatment outcome.

## Supporting information

S1 Protocol MRI velocity measurement protocol. (PDF)
