# Supplementary material for: Phase-contrast MRI versus numerical simulation to quantify hemodynamical changes in cerebral aneurysms after flow diverter treatment
Source: PLoS One. 2018 Jan 5;13(1):e0190696. doi: 10.1371/journal.pone.0190696 (PMC5755883; doi:10.1371/journal.pone.0190696)
Supplement: S1 Protocol — (PDF) [file pone.0190696.s001.pdf]

Phase-Contrast MRI versus Numerical Simulation to Quantify Hemodynamical Changes in Cerebral Aneurysms after Flow Diverter Treatment

Philipp Arnold,Sergey Sindeev,Sergey Frolov,Sascha Prothmann,Dieter Liepsch,Andrea Balasso,Philipp Berg,Stephan Kaczmarz,Jan Kirschke

Abstract
Cerebral aneurysms are a major risk factor for intracranial bleeding with devastating consequences for the patient. One recently established treatment is the implantation of flow diverters (FD). Methods to predict their treatment success before or directly after implantation are not well investigated yet. The aim of this work was to quantitatively study hemodynamic parameters in patient-specific models of treated cerebral aneurysms and its correlation with the clinical outcome. Hemodynamics were evaluated using both computational fluid dynamics (CFD) and phase contrast (PC) MRI. For preoperative and postoperative distribution of hemodynamic parameters, CFD simulations and PC-MRI velocity measurements showed similar results. In both cases where no occlusion of the aneurysm was observed after six months, a flow reduction of about 30-50% was found, while in the clinically successful case with complete occlusion of the aneurysm after 6 months, the flow reduction was about 80%. No vortex was observed in any of the three models after treatment. The results are in agreement with recent studies suggesting that CFD simulations can predict treatment success or failure already before implantation of an FD and PC-MRI could validate the predicted hemodynamic changes right after implantation of an FD.

Citation: Philipp Arnold,Sergey Sindeev,Sergey Frolov,Sascha Prothmann,Dieter Liepsch,Andrea Balasso,Philipp Berg,Stephan Kaczmarz,Jan KirschkePhase-Contrast MRI versus Numerical Simulation to Quantify Hemodynamical Changes in Cerebral Aneurysms after Flow Diverter Treatment. **protocols.io**
https://www.protocols.io/view/phase-contrast-mri-versus-numerical-simulation-to-kekctcw

Protocol

Data aquisition

1. Twenty three patients treated with a FD at the department of Neuroradiology at the Klinikum rechts der Isar between 2008 and 2014 were screened for availability of pre-treatment high spatial resolution 3D angiographic data. These datasets were available for three patients, who were included in this study and subgrouped regarding their clinical treatment outcome. This study was HIPAA compliant and in line with the local ethical and legislative requirements. Patient consent was not required due to the fully anonymous retrospective analysis.
   Group A consisted of one patient (A01) with a clinically successful treatment. This aneurysm showed no perfusion 6 months after FD placement. Group B included two patients (B01, B02) who showed persistent perfusion of the aneurysm for more than 6 months after treatment.

Creating experimental fluid

2. The experimental fluid was a 58% aqueous glycerol mixture, which exhibits Newtonian fluid behavior. To ensure comparable results the mixture of the 2 components of the experimental fluid was tested before each run in the MRI with a refractometer.

Experiment setup

This is an open access protocol distributed under the terms of the Creative Commons Attribution License, which permits unrestricted use, distribution, and reproduction in any medium, provided the original author and source are credited

3. A 3T Philips Ingenia (Philips Healthcare, Netherlands) was used for the phase-contrast MRI (pcMRI) velocity measurements. To maximize the spatial resolution and the signal-to-noise ratio (SNR) the aneurysm phantoms were placed inside a hand wrist coil.
The flow of the emperimental fluid was created by a computer controlled piston pump connected to a closed circuit of silicone tubes. The piston pump provided a realistic flow and pressure pattern which was monitored using a flow meter as well as two pressure sensors (before/after aneurysm phantom). Before starting the pcMRI measurement the piston pump was started until a stable flow (approx. 160ml/min) with physiological systolic and diastolic pressures (approx. 140/80 mmHg) was reached. The MRI was synchronized to the piston pump using a photo diode connected to the piston pump as external trigger for the MRI.

MRI Settings

4. A 4D phase contrast sequence with a multi-shot 3D Gradient-Echo sequence with a turbo-factor of 5 without SENSE was used for image acquisition. The acquisition parameters were set to: TE = 2.9 ms, TR = 6.1 ms, alpha = 10°. Flow in all 3 spatial directions (ap, rl, cc) were acquired consecutively in one single scan. Total scan duration was 30:44 min.

The quantitative velocity field was reconstructed with corrections for magnetic field inhomogeneities by static measurements. The spatial resolution was RLxAPxFH=0.53x0.53x0.60 mm^3 with a matrix size of 112x112x94 voxels. The temporal resolution was 71 ms. After inspection of the CFD velocity data velocity encoding level (VENC) was set to 100 cm/s for each spatial direction to prevent aliasing in the aneurysm region. Partial volume effects close to the vessel wall were neglected as the drop in magnitude signal for all cases with and without FD was steep and abrupt.

Data analysis

5. The MRI data was saved as DICOM files containing magnitude and phase-contrast information for all 3 directions (FH, AP, RL). The DICOM files were read into MATLAB using the dicomread function. The vessel anatomy was reconstructed by threshold based semi-automatic segmentation of the magnitude data. This was done slice by slice with correction by an experienced physician. Voxels outside the vessel anatomy were set to have no flow in any direction.

This is an open access protocol distributed under the terms of the Creative Commons Attribution License, which permits unrestricted use, distribution, and reproduction in any medium, provided the original author and source are credited
